# Clinical, laboratory, and imaging features of pediatric COVID-19: A systematic review and meta-analysis

**DOI:** 10.1097/MD.0000000000025230

**Published:** 2021-04-16

**Authors:** Kai Qi, Weibiao Zeng, Miao Ye, Li Zheng, Chao Song, Sheng Hu, Chuanhui Duan, Yiping Wei, Jinhua Peng, Wenxiong Zhang, Jianjun Xu

**Affiliations:** a Department of Cardiothoracic Surgery, The Second Affiliated Hospital of Nanchang University; b Medical College of Nanchang University; c Department of Gastroenterology Medicine, The Third Affiliated Hospital of Nanchang University, Nanchang, China.

**Keywords:** children, clinical features, coronavirus disease 2019, severe acute respiratory syndrome coronavirus 2

## Abstract

Pediatric cases of coronavirus disease 2019 (COVID-19) have been reported. This meta-analysis was aimed at describing the clinical, laboratory, and imaging characteristics of children with COVID-19 based on published data of pediatric COVID-19 cases.

Search of PubMed, Embase, Web of Sciences, Science Direct, and Google Scholar for articles published until December 14, 2020, that described the clinical, laboratory, and imaging features of children with COVID-19. Data were extracted independently by 2 authors. Random-effects meta-analysis models were used to report pooled results.

Clinical data from 2874 children with COVID-19 from 37 articles were finally included for quantitative analyses. Fever (48.5%, 95% CI: 41.4%–55.6%) and cough (40.6%, 95% CI: 33.9%–47.5%) were the most common symptoms; asymptomatic infection and severe cases, respectively, accounted for 27.7% (95% CI: 19.7%–36.4%) patients and 1.1% of the 1933 patients included. Laboratory tests showed 5.5% (95% CI: 2.8%–8.9%) of the patients had lymphopenia. The pooled prevalence of leukopenia was 7.3% (95% CI: 3.4%–12.2%), and the C-reactive protein level was high in 14.0% (95% CI: 6.8%–22.8%). Chest computed tomography showed unilateral and bilateral lesions, and ground-glass opacity in 29.4% (95% CI: 24.8%–34.3%) and 24.7% (95% CI: 18.2%–31.6%), and 32.9% (95% CI: 25.3%–40.9%), respectively, and normal in approximately 36.0% (95% CI: 27.7%–44.7%).

We found that children with COVID-19 had relatively mild disease, with quite a lot of asymptomatic infections and low rate of severe illness. Data from more regions are needed to determine the prevention and treatment strategies for children with COVID-19.

## 1 Introduction

Coronavirus disease 2019 (COVID-19) is a type of atypical pneumonia that broke out in December 2019, the causative pathogen of which was isolated and named severe acute respiratory syndrome coronavirus 2 (SARS-CoV-2). Due to the rapid spread of COVID-19, the World Health Organization (WHO) has declared the disease a public health emergency of international concern.^[1]^ Although the WHO and countries across the world have issued relevant prevention guidelines,^[2]^ 199 million people in more than 200 countries have been infected, and it seems that the trend of an increase in infections will continue for a long time.

The SARS-CoV-2 belongs to betacoronavirus genus of viruses, to which the severe acute respiratory syndrome and Middle East respiratory syndrome viruses belong as well.^[3]^ Previous studies have shown that COVID-19 can be transmitted from human to human through droplets and contact transmission,^[4]^ the main susceptible population are individuals aged higher than 50 years,^[5]^ the main symptoms are fever and cough, followed by myalgia, headache, and fatigue, and laboratory tests usually show lymphopenia and leukocytosis. Most of the infected people are mildly ill, but severely ill patients can deteriorate and develop a variety of serious complications and might even death.^[6,7]^

Some cases of infection in children have also been reported,^[8]^ which overturned the previous conjecture that children were not susceptible to COVID-19. These sporadic reports indicated that the characteristics of COVID-19 in children differed from those of the disease in adults. For example, the symptoms of pediatric COVID-19 are mild, the decrease in lymphocytes is not obvious and severe cases are rare. However, due to the low incidence of pediatric COVID-19 and the scattered locations of the cases, the present research on pediatric COVID-19 is insufficient. Moreover, many clinical characteristics of pediatric COVID-19 were not fully understood for the limited sample sizes of these studies, and a consensus was not reached,^[9]^ for example, previous studies have shown that the incidence of severe COVID-19 in children is lower than that in adults, however, it was difficult to obtain reliable quantitative data on the incidence of severe COVID-19.

Meta-analyses are usually performed to combine data from randomized clinical trials. However, in many instances, randomized clinical trials are not feasible and only observational studies can be performed (e.g., in the case of Zika virus infection).^[10]^ Therefore, meta-analyses combining data from observational studies are widely conducted to provide more precise estimates of the effects of treatments or risk factors for diseases.^[11]^ We conducted a systematic review and meta-analysis to describe the clinical, laboratory, and imaging characteristics of children with COVID-19.

## 2 Methods

### 2.1 Protocol and registration

This review protocol followed the recommendations established in the MOOSE statement and has been registered in the PROSPERO database (registration number CRD42020180827).^[12]^

### 2.2 Data sources and search strategy

PubMed, Embase, Web of Sciences, Science Direct, and Google Scholar were searched for articles published until December 14, 2020. The following search terms were used: “COVID-19,” “2019 novel coronavirus disease,” “COVID-19 pandemic,” “SARS-CoV-2 infection,” “COVID-19 virus disease,” “2019 novel coronavirus infection,” “2019-nCoV infection,” “coronavirus disease 2019,” “coronavirus disease-19,”“2019-nCoV disease,” “COVID-19 virus infection” and “pediatric,” “child,’ and “children.” No language restrictions were set.

### 2.3 Inclusion and exclusion criteria

Studies were considered eligible if the subjects included pediatric COVID-19 patients (age range: 0–18 years) diagnosed by real-time RT-PCR, and reported at least 1 of the clinical features, laboratory text, imaging characteristics, were included in the meta-analysis. Case reports, review articles, opinion articles, and letters lacking available data were excluded.

### 2.4 Study selection

**Figure 1:**
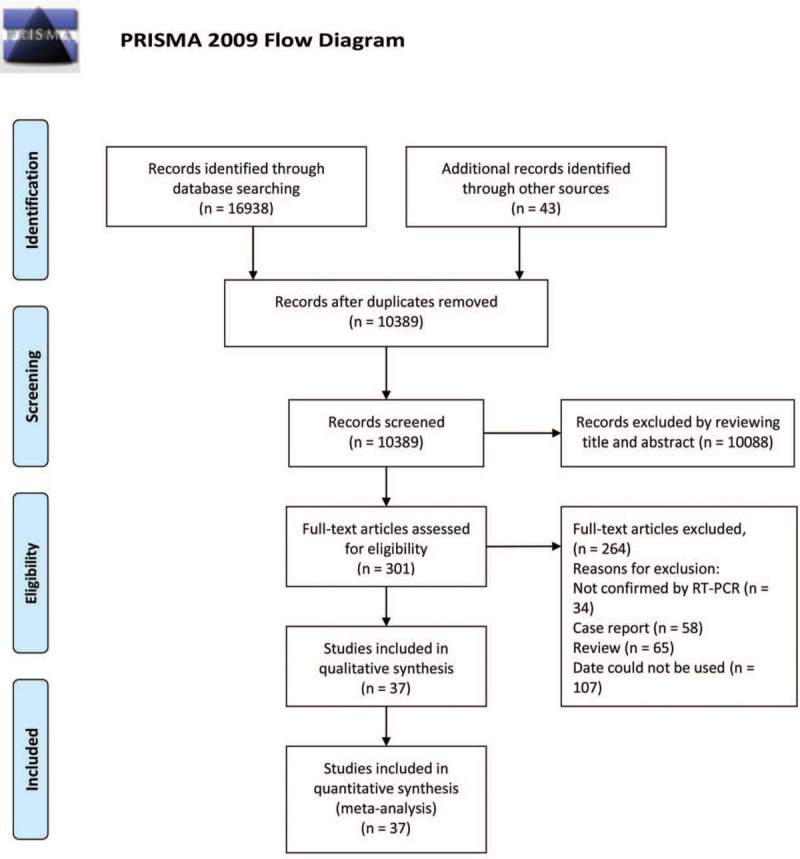
Flow diagram of papers screened and included.

Two reviewers (ZL and SC) browsed the titles and abstracts to filter the search results, followed by the reading of the full text of the articles to determine whether they met the inclusion and exclusion criteria (Fig. 1), different opinions were resolved through consulted and discussed. When the same patient was reported at the same time in 2 articles, we performed statistical analyses with more complete information. References of included articles were also reviewed.

### 2.5 Data collection process and data items

The following features were extracted by 2 investigators using a standardized data extraction form (ZL and SC): country/region, number of reported cases, number of severe cases, patient age, number of asymptomatic cases, history of exposure, clinical characteristics (e.g., fever and cough), laboratory test results (e.g., lymphocytopenia and biochemistry results), and chest computed tomography (CT) findings. All data were checked by a third researcher (XJJ).

### 2.6 Study quality evaluation

Two authors (Zwb and QK) screened and evaluated the literature independently. The quality of the case series was graded using The Joanna Briggs Institute (JBI) critical appraisal checklist.^[13]^ The quality assessment method for descriptive/case series studies recommended by the JBI Reviewer's Manual includes nine quality items, which are judged as “yes,” “no,” “unclear,” or “not applied” for each question. Cross-sectional studies and cohort studies were graded using the Newcastle–Ottawa Scale (NOS).^[14]^

### 2.7 Statistical analysis

The meta-analyses were performed using Stata 15.0, metaprop command. Pooled prevalence and their 95% CIs were used to summarize the weighted effect sizes for each grouping variable in the study by using the binary random-effects model (the weighting took into consideration the sample sizes of the individual studies). Heterogeneity was evaluated using Cochran's Q statistic, the *I*^2^ index, and the tau-squared test. Sensitivity analysis was performed on the basis of outlier data. Funnel plot and Egger regression test were used to evaluate publication biases.

## 3 Results

### 3.1 Study selection and characteristics

A total of 16,938 articles were retrieved by the search strategy, of which 10,389 articles remained after deleting duplicate studies. After screening the abstracts and titles, 301 articles were selected for full-text assessment. Among them, 58 articles were case report, 65 were reviews and 107 were excluded for not showing confirmation analyses by RT-PCR or because of lack of available data. A total of 37 articles, with a total of 2874 patients, were finally included in the quantitative meta-analysis, consisting of 18 cross-sectional studies, 4 cohort studies, and 15 case series.^[8,15–50]^ Twenty seven of the selected studies were from China and 10 were from abroad. Table 1 shows the main characteristics of these included studies. A total of 30 variables were quantitatively synthesized in this meta-analysis (Shown in Table 2).

**Table 1:** Characteristics of the included studies on pediatric COVID-19.

Author	Study type	Region	Year	N	Age	Sex (Male)	Quality score
CDC	Cross-sectional	The U.S.A	2020	291	Range: 0–17 yr	NA	8^∗^
Chen et al	Cross-sectional	China	2020	12	median: 14.5 yr	6	8^∗^
Dong et al	Cross-sectional	China	2020	728	Range: 2–13 yr	418	8^∗^
Du et al	Cross-sectional	China	2020	14	median: 6.2 yr	6	7^∗^
Han et al	Cross-sectional	China	2020	7	Range: 2–13 yr	4	7^∗^
Liang et al	Cross-sectional	China	2020	9	Range: 1–9 yr	3	8^∗^
Liu et al	Cross-sectional	China	2020	4	Range: 1–9 yr	2	8^∗^
Qiu et al	Cross-sectional	China	2020	36	mean: 8 3 yr	23	9^∗^
Wang et al	Cross-sectional	China	2020	31	Range: 1–17 yr	15	8^∗^
Xia et al	Cross-sectional	China	2020	20	median: 2 yr	13	9∗
Xie et al	Cross-sectional	China	2020	13	Range: 10–18 yr	7	9^∗^
Zheng et al	Cross-sectional	China	2020	25	Range: 1–14 yr	14	9^∗^
Zhu et al	Cross-sectional	China	2020	10	Range: 1–17 yr	5	9^∗^
Ma et al	Cross-sectional	China	2020	50	Range: 0–17 yr	28	9^∗^
Korkmaz et al	Cross-sectional	Turkey	2020	81	median: 9.5 yr	48	8^∗^
Wu et al	Cross-sectional	China	2020	74	median: 6 yr	44	9^∗^
Du et al	Cross-sectional	China	2020	182	median: 6 years	120	9^∗^
Parri et al	Cross-sectional	Italy	2020	130	median: 6 yr	73	8^∗^
Bo Li et al	Case series	China	2020	22	mean: 8 yr	12	5/9^†^
Cai et al	Case series	China	2020	10	Range: 1–11 yr	4	5/9^†^
Feng et al	Case series	China	2020	15	Range: 4–14 yr	5	6/9^†^
Li et al	Case series	China	2020	5	Range: 1–6 yr	4	7/9^†^
Lu et al	Case series	China	2020	171	mean: 6.7 yrs	104	7/9^†^
Shen et al	Case series	China	2020	9	Range: 1–12 yr	3	6/9^†^
Tan et al	Case series	China	2020	10	Range: 1–11 yr	3	7/9^†^
Xu et al	Case series	China	2020	10	Range: 1–17 yr	6	8/9^†^
Zhou et al	Case series	China	2020	9	Range: 1–3 yr	4	7/9^†^
Steinberger et al	Case series	China	2020	30	median: 10 years	15	7/9^†^
Wu et al	Case series	China	2020	157	median: 7 yr	60	7/9^†^
Ma et al	Cohort study	China	2020	216	median: 7.25 yr	134	9^∗^
Han et al	Cohort study	Korea	2020	91	median: 11 yr	53	9^∗^
Kilani et al	Cohort study	Jordan	2020	61	median: 6 yr	37	7^∗^
Pablo et al	Case series	Worldwide	2020	91	median: 6.1 yr	49	6/9^†^
Afshin et al	Case series	Iran	2020	27	mean: 4.7 ± 4.16 yr	10	5/9^†^
Fakiri et al	Case series	Morocco	2020	74	median: 7 yr	34	5/9^†^
Danah et al	Cohort study	Kuwait	2020	134	median: 8.8 yr	74	7^∗^
Mamishi et al	Case series	Iran	2020	24	median: 6 yr	11	5/9^†^

**Table 2:** Meta-analysis outcomes of clinical manifestations, laboratory findings and CT imaging findings of pediatric COVID-19 patients^∗^.

Variable	Number of studies	Number of patients	Prevalence %	95% CI	Q^†^	*I*^2^^‡^	*t*^2^^§^	*P*
Clinical manifestations								
Fever	36	2146	48.5	41.4–55.6	293.28	88.07	0.13	<.001
Cough	35	2116	40.6	33.9–47.5	260.34	86.94	0.11	<.001
Dyspnea	21	1284	7.0	2.3–13.5	207.53	90.36	0.16	<.001
Myalgia	13	985	7.1	2.0–14.2	107.13	88. 8	0.12	<.001
Runny nose	16	1295	11.0	6.9–15.8	64.81	76.86	0.04	<.001
Sore throat	23	1310	6.8	2.8–12.0	149.16	85.25	0.11	<.001
Headache	13	947	9.2	4.1–15.7	83.75	85.67	0.09	<.001
Abdominal pain	12	1047	3.6	1.7–6.0	21.44	48.7	0.01	.029
Nausea/Vomiting	17	1388	5.7	3.6–8.1	32.38	50.59	0.01	.009
Diarrhea	23	1571	7.2	5.0–9.5	40.25	45.34	0.01	.010
Fatigue	12	814	5.7	2.7–9.4	23.95	54.08	0.02	.013
Asymptomatic	28	2121	27.7	19.7–36.4	348.92	92.26	0.18	<.001
Severe cases	20	1933	1.1	0–2.9	71.86	73.56	0.03	<.001
Laboratory findings								
Lymphocytosis	15	542	8.5	3.1–15.4	52.01	73.08	0.08	<.001
Lymphopenia	25	1303	5.5	2.8–8.9	80.43	70.16	0.05	<.001
Leukocytosis	19	573	3.5	0.6–8.1	47.49	62.1	0.06	<.001
Leukopenia	27	1320	7.3	3.4–12.2	146.21	82.22	0.10	<.001
High CRP	24	1043	14.0	6.8–22.8	215.78	89.34	0.20	<.001
High LDH	14	540	17.4	7.8–29.3	100.38	87.05	0.19	<.001
High ALT	21	1035	6.2	2.7–10.6	81.36	75.42	0.07	<.001
High AST	19	997	12.3	7.5–17.8	69.92	74.26	0.06	<.001
High CK-MB	8	488	43	25.4–61.5	84.48	91.71	0.21	<.001
High ESR	10	247	29.7	10.0–53.3	79.51	88.68	0.36	<.001
D-dimer increase	11	508	9.3	5.1–14.3	16.38	38.94	0.02	.089
Procalcitonin increase	15	850	22.2	9.6–37.7	277.41	94.95	0.35	<.001
CT imaging findings								
Normal	28	1096	36.0	27.7–44.7	173.06	84.40	0.15	<.001
Unilateral lesion	18	872	29.4	24.8–34.3	26.00	34.61	0.01	0.075
Bilateral lesion	18	872	24.7	18.2–31.6	56.56	69.94	0.05	<.001
Ground-glass opacity	22	935	32.9	25.3–40.9	98.06	78.59	0.09	<.001
Contact with a confirmed case	26	1354	93.6	88.9–97.3	146.42	82.93	0.10	<.001

### 3.2 Quality assessment

According to the JBI critical appraisal checklist, the quality of the 15 case-series studies were evaluated. There were 5 scores of 5 points, 9 scores of 6 to 7 points, and 1 score of 8 points. Eighteen cross-sectional studies and 4 cohort studies conducted quality evaluation based on NOS scores, 18 of which were high-quality studies of 8 to 9 points, and 4 was medium-quality study of 7 points. (See Table S1 and S2, Supplemental Content, which shown the details of quality assessment of the articles).

### 3.3 Clinical characteristics

As shown in Table 2, fever (48.5%, 95% CI: 41.4–55.6%) and cough (40.6%, 95% CI: 33.9%–47.5%) were the most common symptoms, almost all studies have counted these 2 symptoms. Runny nose (11.0%, 95% CI: 6.9%–15.8%), headache (9.2%, 95% CI: 4.1%–15.7%), sore throat (6.8%, 95% CI: 2.8%–12.0%) were reported in 16, 13, and 23 studies, respectively. Other symptoms are relatively rare and few studies have been reported. Asymptomatic infection and severe cases, respectively, accounted for 27.7% (95% CI: 19.7%–36.4%) and 1.1% (95% CI: 0%–2.9%) of patients. (See Table S3 and Figure S2, Supplemental Content, which shown details of results of clinical characteristics of pediatric COVID-19 patients).

### 3.4 Laboratory findings

As shown in Table 2, only 5.5% (95% CI: 2.8%–8.9%) of children with COVID-19 showed lymphopenia in laboratory tests, which is very common in adult COVID-19. The pooled prevalence of leukopenia in pediatric COVID-19 patients was estimated to be 7.3% (95% CI: 3.4–12.2%), while the corresponding values for high C-reactive protein level, high LDH level, high creatine kinase MB level, high AST level, and high erythrocyte sedimentation rate were 14.0% (95% CI: 6.8%–22.8%), 17.4% (95% CI: 7.8%–29.3%), 43% (95% CI: 25.4%–61.5%), 12.3% (95% CI: 7.5%–17.8%), and 29.7% (95% CI: 10.0%–53.3%), respectively. Abnormal results in laboratory tests for COVID-19 in children were not as common as those in adults. (See Table S4 and Figure S2, Supplemental Content, which shown details of results of laboratory characteristics of pediatric COVID-19 patients).

### 3.5 Imaging and demographic characteristics

In the chest CT imaging data summarized in Table 2, about 36.0% (95% CI: 27.7–44.7%) of the pediatric COVID-19 patients did not show abnormal imaging findings. Among patients with abnormal imaging findings, unilateral lesions, bilateral lesions, and ground-glass opacity accounted for 29.4% (95% CI: 24.8%–34.3%), 24.7% (95% CI: 18.2%–31.6%) and 32.9% (95% CI: 25.3%–40.9%), of the cases. In assessments of demographic characteristics, the results showed that 93.6% (95% CI: 88.9%–97.3%) of the pediatric COVID-19 patients had contact with a confirmed case. (See Table S5 and Figure S2, Supplemental Content, which shown details of results of Imaging characteristics, contact history, and severe cases of pediatric COVID-19 patients).

### 3.6 Publication bias

The funnel plot (standard error of fever) showed no obvious publication bias (see Figure S1, Content, Funnel-plot for publication bias of fever of pediatric COVID-19 patients), and the findings of Egger test also proved this conclusion (*P* = .428).

## 4 Discussion

As of September 21, 2020, there were 1 998 897confirmed cases of COVID-19 and 954 417 deaths attributable to the disease worldwide. Children were once considered not susceptible to COVID-19, but according to survey data released by the centers for disease control (CDC) on April 2, the United States recorded 2572 cases (1.7%) of children with COVID-19 under the age of 18 years.^[15]^ A survey of 72,314 patients in China showed that children accounted for about 2% of the total number of cases.^[51]^ In addition, children have atypical symptoms of infection and are unable to clearly describe their health status or exposure history, which poses serious challenges to the protection, diagnosis, and treatment of this population.^[52]^ The increase in the number of children's infections and the particularity of clinical manifestations in pediatric COVID-19 infections necessitated a more comprehensive understanding of the clinical, laboratory, and imaging features of pediatric COVID-19.

In this systematic review and random-effects meta-analysis, we tried to conduct a comprehensive quantitative analysis of the published clinical data for COVID-19 in children to obtain clinical features with a larger sample size and more reliable results. Our results are robust due to the pooling of results after combining all the studies, which showed acceptable heterogeneity in forest plots for each of the variables (Table 2). Due to the small sample size of most included studies, the data do not conform to the normal distribution. Fixed-effect meta-analyses are inappropriate in such cases, so we used random-effects analyses.^[53]^

Most children with COVID-19 have mild clinical symptoms, and some infected patients show no obvious clinical manifestations. Lu et al reported the clinical information of 171 children with COVID-19 admitted to Wuhan Children's Hospital. Most of the patients had mild manifestations, with 15.8% (27/171) showing asymptomatic infection and only 3 showing severe disease.^[28]^ Our results also show these characteristics in children with COVID-19. Fever is the most common symptom in children with COVID-19, but its incidence (50%, 95% CI: 43%–56%) was significantly lower than that in adults (about 70%).^[54,55]^ The second most common infection symptoms were cough (40.6%, 95% CI: 33.9%–47.5%), myalgia (7.1%, 95% CI: 2.0%–14.2%), and sore throat (6.8%, 95% CI: 2.8%–12.0%). Notably, the probability of gastrointestinal symptoms in children was more than 15%, which suggested that gastrointestinal symptoms should receive special attention when diagnosing children with COVID-19.^[56]^ Our results showed that children with severe illness account for only 1.1% of all hospitalized children, and all of these patients had severe underlying diseases. In contrast, the reported critical illness rate for adults is about 3% to 15%.^[57]^ Corresponding to the lower rate of severe illness in children with COVID-19, about 27.7% of children COVID-19 were asymptomatic, while a study of 44672 diagnosed patients of all ages from Chinese Novel Coronavirus Pneumonia Emergency Response Epidemiology Team showed that asymptomatic infections account for only 1.2%.^[58]^ Only 48.5% of children with COVID-19 showed symptoms of fever and 27.7% of the infections were asymptomatic, indicating the possibility of numerous omissions when screening pediatric COVID-19 patients by monitoring body temperature.

Lymphocytopenia, which is an important feature of adult COVID-19 and is considered to be one of the indicators to predict the severity of the disease was rare in children with COVID-19 (5.5%, 95% CI: 2.8%–8.9%).^[59]^ Previous studies have shown that SARS-CoV2 will induce a series of immune responses after entering the body, which then induces inflammatory storms, leading to increases in inflammatory indicators and a decline in lymphocyte counts.^[60,61]^ In our results, leukopenia, high C-reactive protein levels, high erythrocyte sedimentation rate, and high ALT levels were reported less frequently, which indicated that the immune responses of children with COVID-19 were weak. The imaging results showed that about 36% of children's chest CT scans had no imaging abnormalities, and many showed only slight local invasion, indicating that children with COVID-19 had only mild lung injury and a good prognosis.^[62]^

The data for clinical symptoms along with the findings of laboratory tests and imaging examinations of children with COVID-19 in this study indicate that the conditions of children with COVID-19 generally seem to be relatively mild. The possible reasons are as follows: first, children have healthier airways because they are not exposed to cigarette smoke and air pollution for long periods, both of which are thought to contribute to COVID-19.^[62,63]^ Second, many other kinds of viruses are found in children's lungs and respiratory tract, which can restrict the growth of SARA-CoV2 through direct interaction and competition between viruses.^[64]^ Third, the number of mature angiotensin-converting enzyme-2 (ACE2) receptors in children's lungs is lower than that in adults.^[65,66]^ SARS-CoV2 uses the ACE2 receptors on the cell surface to enter human airway epithelial cells, and the limited number of ACE2 receptors enhance children's resistance to SARS-CoV2.^[67]^ Fourth, the immune system of children is not yet mature. The SARS-CoV2 infection will not produce a large number of inflammatory factors, reducing the damage of autoimmunity to the lungs, heart, liver, and other organs, and the possibility of occurrence of an inflammatory storm, which is an important mechanism leading to the death of patients with severe disease.^[68,69]^ Therefore, lymphocytes in children with COVID-19 rarely decline significantly, and the levels of inflammation indicators such as C-reactive protein are usually normal or transiently elevated. Fifth, since children indulge in relatively limited outdoor activities, they are usually infected by their families, and the virulence of these second- or third-generation infections may be lower in children.^[49]^ The combined effect of these factors leads to a mild condition in children with COVID-19. However, these children are still contagious, and because of their concealed, mild, and asymptomatic disease, pediatric COVID-19 patients may be a key link in the community transmission of SARS-CoV2. Early detection and treatment of children with COVID-19 are of great significance to prevent the spread of SARS-CoV2.^[8]^

At present, Chang et al has made the 1 preliminary description of the clinical symptoms of children with COVID-19 through pool clinical data,^[51]^ which was basically consistent with our conclusion. However, the number of articles included by Chang et al was limited, there were only 9 early studies with a small sample size, and their study did not collect data from laboratory tests, the description of the clinical characteristics of children with COVID-19 was not comprehensive enough. In addition, there are some studies had analyzed the clinical characteristics of COVID-19 in adults using si2milar methods.^[70]^

Our conclusions are robust, but there is still some heterogeneity, which can be attributed to the following reasons: First, all of the included studies were retrospective observational studies, and there were no randomized controlled studies. Second, because of the low incidence of COVID-19 in children, the sample size of many studies was small. Third, although all studies included were on children, the age ranges of children in each study were different. Fourth, the criteria for judging some indicators, such as headache and bilateral lesions, in each study may be different.

### 4.1 Limitations

Some limitations of this study must be pointed out. First, because of the low mortality rate of children with COVID-19, we did not collect enough data for synthesis analysis. Only a few large-scale statistical studies provided a small number of deaths. Of the 149,082 confirmed cases released by the CDC in the United States on April 2nd, only 3 deaths were recorded among children.^[15]^ Data published on March 18 in Italy reported that of the 22,512 Italian cases of COVID-19, 1.2% involved children, with no mortalities.^[71]^ Second, most of the studies included in the study are from China, with only 1 from the United States, 1 from Turkey and 1 from Italy, and more data from COVID-19 in children from other countries are needed to obtain more comprehensive conclusions. Third, to comprehensively describe the characteristics of COVID-19 in children, this study included a large number of articles, resulting in many sources of heterogeneity and the lack of effective means to reduce heterogeneity, but yielding a more comprehensive analysis. Therefore, our conclusions need to be verified by more rigorous large-sample studies.

## 5 Conclusions

This meta-analysis found that children with COVID-19 have relatively mild disease, with quite a lot of asymptomatic infections and a very low rate of severe illness. The results of laboratory tests were only slightly abnormal. Chest CT showed no obvious abnormality in quite a part of cases, and the scope of lung injury was limited. Data from studies on children with COVID-19 in more regions are needed to determine the best prevention and treatment strategies for pediatric COVID-19 cases.

## Author contributions

XJJ and WYP designed the study; QK and ZWB interpreted data and wrote the manuscript; ZL and SC screened and extracted data; HS and DCH conducted statistical analyses; PJH and ZWX reviewed the results and made critical comments on the manuscript: All authors approved the final version of the manuscript.

**Data curation:** Chao Song.

**Formal analysis:** Li Zheng.

**Investigation:** Miao Ye, Sheng Hu.

**Methodology:** Chuanhui Duan.

**Project administration:** Yiping Wei, Jianjun Xu.

**Supervision:** Jinhua Peng.

**Visualization:** Wenxiong Zhang.

**Writing – original draft:** Kai Qi, Weibiao Zeng.

## Supplementary Material

Supplemental Digital Content

## Supplementary Material

Supplemental Digital Content

## Supplementary Material

Supplemental Digital Content

## Supplementary Material

Supplemental Digital Content

## Supplementary Material

Supplemental Digital Content

## Supplementary Material

Supplemental Digital Content

## Supplementary Material

Supplemental Digital Content

## Supplementary Material

Supplemental Digital Content

## Supplementary Material

Supplemental Digital Content

## Supplementary Material

Supplemental Digital Content
