# Supplementary material for: Clinical, laboratory, and imaging features of pediatric COVID-19: A systematic review and meta-analysis
Source: Medicine (Baltimore). 2021 Apr 16;100(15):e25230. doi: 10.1097/MD.0000000000025230 (PMC8052054; doi:10.1097/MD.0000000000025230)

**Figure S1.** Funnel-plot for publication bias of fever of pediatric COVID-19 patients.

Funnel plot (standard error of fever) showed no obvious publication bias.


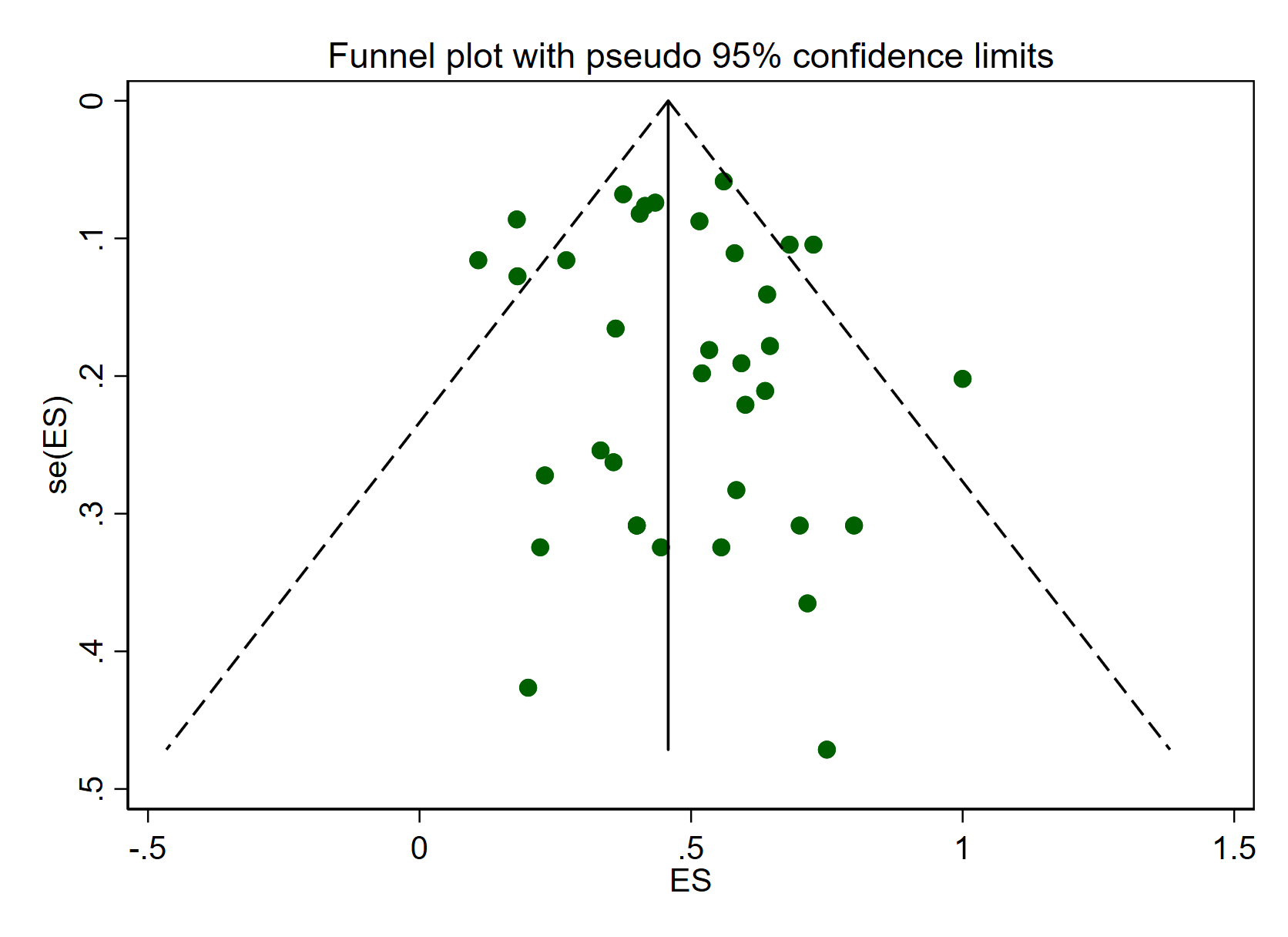

Supplement: Supplemental Digital Content [file medi-100-e25230-s001.doc]
