# Supplementary material for: Clinical, laboratory, and imaging features of pediatric COVID-19: A systematic review and meta-analysis
Source: Medicine (Baltimore). 2021 Apr 16;100(15):e25230. doi: 10.1097/MD.0000000000025230 (PMC8052054; doi:10.1097/MD.0000000000025230)
Supplement: Supplemental Digital Content [file medi-100-e25230-s002.doc]

**Figure S2.** Forest plots of clinical manifestations, laboratory findings and CT imaging findings of pediatric COVID-19 patients.

**Figure S2a**: 1, Fever; 2, Cough; 3, Dyspnea; 4, Myalgia; 5, Runny nose; 6, Sore throat; 7, Headache; 8, Abdominal pain
