# Supplementary material for: Clinical, laboratory, and imaging features of pediatric COVID-19: A systematic review and meta-analysis
Source: Medicine (Baltimore). 2021 Apr 16;100(15):e25230. doi: 10.1097/MD.0000000000025230 (PMC8052054; doi:10.1097/MD.0000000000025230)
Supplement: Supplemental Digital Content [file medi-100-e25230-s003.doc]

**Figure S2b**: 9, Nausea/Vomiting; 10, Fatigue; 11, Diarrhea; 12, Asymptomatic; 13, Severe; 14, Lymphocytosis; 15, Lymphocytopenia; 16, Leukopenia
