# Supplementary material for: Clinical, laboratory, and imaging features of pediatric COVID-19: A systematic review and meta-analysis
Source: Medicine (Baltimore). 2021 Apr 16;100(15):e25230. doi: 10.1097/MD.0000000000025230 (PMC8052054; doi:10.1097/MD.0000000000025230)
Supplement: Supplemental Digital Content [file medi-100-e25230-s004.doc]

**Figure S2c**: 17, Leukocytosis; 18, High CRP; 19, High LDH; 20, Procalcitonin increase; 21, High ALT; 22, High AST; 23, D-dimer increase; 24, High CK-MB
