# Supplementary material for: Clinical, laboratory, and imaging features of pediatric COVID-19: A systematic review and meta-analysis
Source: Medicine (Baltimore). 2021 Apr 16;100(15):e25230. doi: 10.1097/MD.0000000000025230 (PMC8052054; doi:10.1097/MD.0000000000025230)
Supplement: Supplemental Digital Content [file medi-100-e25230-s005.doc]

**Figure S2d**: 25, High ESR; 26, Ground-glass opacity; 27, Bilateral lesions; 28, Unilateral lesion; 29, Normal; 30, Contact with a confirmed case.
