# Supplementary material for: Clinical, laboratory, and imaging features of pediatric COVID-19: A systematic review and meta-analysis
Source: Medicine (Baltimore). 2021 Apr 16;100(15):e25230. doi: 10.1097/MD.0000000000025230 (PMC8052054; doi:10.1097/MD.0000000000025230)
Supplement: Supplemental Digital Content [file medi-100-e25230-s006.docx]

**Table S1.** Joanna Briggs Institute quality assessment scale for case series.

| Author | Year | Q1 | Q2 | Q3 | Q4 | Q5 |  | Q6 | Q7 | Q8 | Q9 | Total |
| --- | --- | --- | --- | --- | --- | --- | --- | --- | --- | --- | --- | --- |
| Cai et al. | 2020 | U | Y | NA | Y | NA |  | Y | NA | Y | Y | 5\9 |
| Feng et al. | 2020 | U | Y | Y | Y | NA |  | Y | NA | Y | Y | 6\9 |
| Bo Li et al. | 2020 | U | Y | U | Y | NA |  | Y | NA | Y | Y | 5\9 |
| Xu et al. | 2020 | Y | Y | Y | Y | NA |  | Y | Y | Y | Y | 8\9 |
| Tan et al. | 2020 | Y | Y | NA | Y | NA |  | Y | NA | Y | Y | 7\9 |
| Lu et al. | 2020 | Y | Y | Y | Y | NA |  | Y | NA | Y | Y | 7\9 |
| Li et al. | 2020 | Y | Y | NA | Y | NA |  | Y | NA | Y | Y | 7\9 |
| Shen et al. | 2020 | Y | Y | U | Y | NA |  | Y | NA | Y | Y | 6\9 |
| Zhou et al. | 2020 | Y | Y | Y | Y | NA |  | Y | NA | Y | Y | 7\9 |
| Steinberger et al. | 2020 | Y | Y | Y | Y | NA |  | Y | NA | Y | Y | 7\9 |
| Wu et al. | 2020 | Y | Y | Y | Y | NA |  | Y | NA | Y | Y | 7\9 |
| Pablo et al. | 2020 | U | Y | Y | Y | NA |  | Y | NA | Y | Y | 6\9 |
| Afshin et al. | 2020 | U | Y | U | Y | NA |  | Y | NA | Y | Y | 5\9 |
| Fakiri et al. | 2020 | U | Y | NA | Y | NA |  | Y | NA | Y | Y | 5\9 |
| Mamishi et al. | 2020 | U | Y | NA | Y | NA |  | Y | NA | Y | Y | 5\9 |

N, no.; NA, not applicable; U, unclear; Y, yes.
