# Supplementary material for: Clinical, laboratory, and imaging features of pediatric COVID-19: A systematic review and meta-analysis
Source: Medicine (Baltimore). 2021 Apr 16;100(15):e25230. doi: 10.1097/MD.0000000000025230 (PMC8052054; doi:10.1097/MD.0000000000025230)
Supplement: Supplemental Digital Content [file medi-100-e25230-s007.docx]

| **Table S2.** Newcastle-Ottawa quality assessment scale for cross-sectional studies. | | | | | | |
| --- | --- | --- | --- | --- | --- | --- |
| Author | Year | Selection | Comparability | Exposure/Outcome | Total Score |  |
| Dong et al. | 2020 | **** | * | *** | ******** | |
| CDC | 2020 | **** | ** | ** | ******** | |
| Wang et al. | 2020 | **** | * | *** | ******** | |
| Chen et al. | 2020 | **** | ** | ** | ******** | |
| Du et al. | 2020 | *** | ** | ** | ******* | |
| Xia et al. | 2020 | **** | * | *** | ******** | |
| Zheng et al. | 2020 | **** | ** | *** | ********* | |
| Zhu et al. | 2020 | **** | ** | *** | ********* | |
| Liu et al. | 2020 | **** | ** | ** | ******** | |
| Liang et al. | 2020 | **** | ** | ** | ******** | |
| Xie et al. | 2020 | **** | ** | *** | ********* | |
| Han et al. | 2020 | *** | ** | *** | ******** | |
| Qiu et al. | 2020 | **** | ** | *** | ********* | |
| Ma et al. | 2020 | **** | ** | *** | ********* | |
| Korkmaz et al. | 2020 | **** | ** | ** | ******** | |
| Wu et al. | 2020 | **** | ** | *** | ********* | |
| Du et al. | 2020 | **** | ** | *** | ********* | |
| Parri et al. | 2020 | *** | ** | *** | ******** | |
| Ma et al. | 2020 | **** | ** | *** | ********* | |
| Han et al. | 2020 | **** | ** | *** | ********* | |
| Kilani et al. | 2020 | *** | ** | ** | ******* | |
| Danah et al. | 2020 | *** | ** | ** | ******* | |
