# Supplementary material for: Clinical, laboratory, and imaging features of pediatric COVID-19: A systematic review and meta-analysis
Source: Medicine (Baltimore). 2021 Apr 16;100(15):e25230. doi: 10.1097/MD.0000000000025230 (PMC8052054; doi:10.1097/MD.0000000000025230)
Supplement: Supplemental Digital Content [file medi-100-e25230-s008.docx]

**Table S3.** Clinical characteristics of pediatric COVID-19 patients of the included studies.

| Author | N | n |  |  |  |  |  |  |  |  |  |  |  |  |  |  |
| --- | --- | --- | --- | --- | --- | --- | --- | --- | --- | --- | --- | --- | --- | --- | --- | --- |
|  |  | Fever | Cough | Dyspnea | Myalgia | Runny nose | Sore throat | Headache | Nausea/  Vomiting | Abdominal pain | Diarrhea | Fatigue | Asymptomatic | Contact history | Severe |  |
|  |  |  |  |  |  |  |  |  |  |  |  |  |  |  |  |  |
| Dong et al. | 728 | NA | NA | NA | NA | NA | NA | NA | NA | NA | NA | NA | 94 | NA | 21 |  |
| CDC | 291 | 163 | 158 | 39 | 66 | 21 | 71 | 81 | 31 | 17 | 37 | NA | NA | NA | NA |  |
| Wang et al | 31 | 20 | 14 | 0 | NA | 2 | 2 | 3 | 2 | NA | 3 | 3 | 4 | 22 | 0 |  |
| Cai et al. | 10 | 8 | 6 | 0 | NA | 4 | 4 | NA | NA | NA | 0 | NA | 0 | 8 | NA |  |
| Chen et al. | 12 | 7 | 9 | NA | NA | NA | NA | NA | NA | NA | 4 | 1 | 2 | 12 | 0 |  |
| Du et al. | 14 | 5 | 4 | 0 | 1 | NA | 1 | 1 | 0 | 0 | 0 | 1 | 8 | NA | 0 |  |
| Feng et al. | 15 | 5 | 1 | NA | NA | 1 | NA | NA | NA | NA | NA | NA | 8 | 15 | 0 |  |
| Bo Li et al. | 22 | 14 | 13 | NA | NA | NA | NA | NA | NA | NA | NA | NA | 2 | NA | NA |  |
| Xu et al. | 10 | 7 | 5 | 0 | NA | 2 | 4 | NA | NA | NA | 3 | NA | 1 | 10 | 0 |  |
| Qiu et al. | 36 | 13 | 7 | 1 | NA | NA | 2 | 2 | 2 | NA | NA | NA | 10 | 32 | 0 |  |
| Tan et al. | 10 | 4 | 3 | NA | NA | NA | NA | NA | 1 | 1 | NA | NA | 2 | 10 | NA |  |
| Xia et al. | 20 | 12 | 13 | NA | NA | 3 | 1 | NA | 2 | NA | 3 | 1 | NA | NA | NA |  |
| Zheng et al. | 25 | 13 | 11 | 2 | NA | NA | NA | NA | 2 | 2 | 3 | NA | NA | 21 | 2 |  |
| Zhu et al. | 10 | 4 | 3 | 0 | NA | NA | 0 | 2 | NA | NA | 0 | NA | 3 | 7 | NA |  |
| Lu et al. | 171 | 71 | 83 | 49 | NA | 22 | NA | NA | 11 | NA | 15 | 13 | 27 | 156 | 3 |  |
| Li et al. | 5 | 1 | 1 | NA | NA | 1 | 1 | NA | NA | NA | NA | NA | 4 | NA | NA |  |
| Liu et al. | 4 | 3 | 3 | NA | NA | NA | NA | NA | NA | NA | NA | 1 | 0 | 4 | NA |  |
| Shen et al. | 9 | 5 | 1 | NA | NA | NA | 1 | NA | NA | NA | 2 | NA | 2 | 9 | 0 |  |
| Liang et al. | 9 | 2 | 1 | 0 | NA | NA | NA | NA | NA | NA | NA | NA | 6 | 9 | 0 |  |
| Xie et al. | 13 | 3 | 4 | NA | 4 | NA | NA | NA | NA | NA | NA | NA | NA | 9 | 0 |  |
| Zhou et al. | 9 | 4 | 2 | 0 | 0 | 1 | 0 | 0 | 0 | 0 | 0 | 0 | 5 | 9 | NA |  |
| Han et al. | 7 | 5 | 5 | 3 | 0 | NA | 1 | NA | NA | NA | NA | NA | NA | 7 | NA |  |
| Ma et al. | 50 | 32 | 22 | NA | 2 | 8 | 1 | NA | NA | 2 | 3 | 2 | 6 | 50 | 2 |  |
| Korkmaz et al. | 81 | 47 | 42 | 2 | 15 | 3 | 7 | 11 | 3 | NA | NA | 15 | 17 | 73 | NA |  |
| Hua et al. | 30 | 16 | NA | 1 | NA | NA | 1 | NA | 0 | NA | 1 | NA | 9 | 29 | NA |  |
| Wu et al. | 74 | 20 | 24 | 2 | 0 | NA | 0 | 2 | NA | NA | 3 | NA | 20 | 65 | 1 |  |
| Du et al. | 182 | 79 | 81 | 2 | NA | NA | NA | NA | 7 | 7 | 9 | 5 | 55 | 178 | NA |  |
| Wu et al. | 148 | 60 | 66 | NA | NA | NA | NA | 5 | NA | NA | NA | NA | 45 | NA | NA |  |
| Parri et al. | 130 | 67 | 38 | NA | NA | 25 | 9 | NA | 15 | NA | 10 | NA | NA | 70 | 20 |  |
| Ma et al. | 216 | 81 | 79 | NA | 4 | 7 | 3 | NA | 7 | 3 | 11 | 10 | NA | 193 | 0 |  |
| Han et al. | 91 | 62 | 66 | 1 | 7 | 24 | 22 | 12 | 6 | 6 | 11 | NA | 20 | 87 | 2 |  |
| Kilani et al. | 61 | 11 | 7 | 2 | 10 | NA | 2 | 12 | NA | 4 | 10 | NA | 27 | NA | NA |  |
| Pablo et al. | 91 | 66 | 50 | 33 | NA | 13 | NA | NA | NA | NA | NA | NA | 5 | NA | 9 |  |
| Afshin et al. | 27 | 16 | 23 | 16 | 0 | NA | 4 | 2 | 4 | NA | 1 | NA | NA | NA | NA |  |
| Fakiri et al. | 74 | 8 | 5 | NA | NA | 5 | 1 | 3 | NA | 1 | 4 | NA | 54 | 74 | 0 |  |
| Danah et al. | 134 | 24 | 32 | NA | NA | NA | NA | NA | NA | NA | NA | NA | 91 | 130 | 0 |  |
| Mamishi et al. | 24 | 24 | 15 | 7 | 4 | NA | 1 | NA | 5 | 5 | 3 | 3 | NA | NA | NA |  |

CDC, CDC COVID-19 Response Team；Contact history, contact with a conﬁrmed case; NA, not available, not reported.
