# Supplementary material for: Clinical, laboratory, and imaging features of pediatric COVID-19: A systematic review and meta-analysis
Source: Medicine (Baltimore). 2021 Apr 16;100(15):e25230. doi: 10.1097/MD.0000000000025230 (PMC8052054; doi:10.1097/MD.0000000000025230)
Supplement: Supplemental Digital Content [file medi-100-e25230-s009.docx]

**Table S4**. Laboratory characteristics of pediatric COVID-19 patients of the included studies.

| Author | N | n |  |  |  |  |  |  |  |  |  |  |  |  |
| --- | --- | --- | --- | --- | --- | --- | --- | --- | --- | --- | --- | --- | --- | --- |
|  |  | Lymphocytopenia | Lymphocytosis | Leukopenia | Leukocytosis | High CRP | High LDH | High ALT | High AST | Procalcitonin increase | D-dimer increase | High CK-MB | High ESR |  |
|  |  |  |  |  |  |  |  |  |  |  |  |  |  |  |
| Wang et al. | 31 | 2 | 4 | 2 | 3 | 3 | 2 | NA | NA | 1 | 2 | NA | 4 |  |
| Cai et al. | 10 | 0 | 1 | 1 | 3 | 3 | 3 | 1 | 2 | 0 | 2 | 5 | NA |  |
| Chen et al. | 12 | 0 | 0 | 0 | 0 | NA | NA | 0 | 0 | NA | NA | NA | NA |  |
| Du et al. | 14 | 1 | NA | 4 | NA | 1 | 7 | 1 | 1 | 5 | 5 | NA | NA |  |
| Xu et al. | 10 | 3 | 2 | 3 | 0 | 3 | 2 | 1 | 2 | 5 | 1 | NA | 3 |  |
| Qiu et al. | 36 | 11 | NA | 7 | NA | 1 | NA | 2 | 3 | 6 | 3 | 11 | NA |  |
| Tan et al. | 10 | 0 | 1 | 0 | 1 | 0 | 0 | 0 | 2 | NA | NA | NA | NA |  |
| Xia et al. | 20 | NA | NA | 4 | 2 | 9 | NA | 5 | NA | 16 | NA | 15 | NA |  |
| Zheng et al. | 25 | 10 | NA | NA | NA | NA | NA | NA | NA | NA | NA | NA | NA |  |
| Zhu et al. | 10 | 0 | 0 | 0 | 0 | 0 | NA | 3 | NA | 0 | NA | NA | NA |  |
| Lu et al. | 171 | 6 | NA | 45 | NA | 33 | NA | 21 | 25 | 105 | 21 | NA | NA |  |
| Li et al. | 5 | NA | NA | 0 | 1 | 1 | NA | NA | NA | NA | NA | NA | NA |  |
| Liu et al. | 4 | 0 | 2 | 1 | 0 | 1 | NA | NA | NA | NA | NA | NA | NA |  |
| Shen et al. | 9 | 0 | 2 | 0 | 1 | 1 | 0 | 0 | 2 | NA | NA | NA | 4 |  |
| Liang et al. | 9 | NA | NA | 2 | 1 | 0 | NA | 0 | 0 | 0 | NA | 6 | 0 |  |
| Xie et al. | 13 | 0 | 0 | 0 | 0 | 0 | NA | NA | NA | NA | NA | NA | NA |  |
| Zhou et al. | 9 | NA | 6 | NA | 2 | 3 | 3 | 0 | 4 | NA | 0 | NA | NA |  |
| Han et al. | 7 | 0 | NA | 0 | 2 | 2 | 2 | 1 | 2 | 3 | 2 | 4 | 3 |  |
| Ma et al. | 50 | 8 | 4 | 19 | 2 | 10 | NA | NA | NA | NA | NA | NA | NA |  |
| Korkmaz et al. | 81 | 4 | NA | 2 | NA | 13 | 14 | 2 | 7 | 3 | 10 | 4 | 9 |  |
| Hua et al. | 30 | 6 | 4 | 3 | 0 | NA | NA | NA | NA | NA | NA | NA | NA |  |
| Wu et al. | 74 | 4 | NA | 4 | NA | 2 | NA | NA | NA | 2 | NA | NA | 6 |  |
| Du et al. | 182 | 7 | 18 | 12 | 17 | 25 | NA | 9 | 24 | 75 | 11 | 84 | NA |  |
| Wu et al. | 148 | 7 | NA | 16 | NA | 48 | 32 | 12 | 25 | 70 | NA | 64 | NA |  |
| Parri et al. | 130 | 3 | NA | NA | NA | NA | NA | 8 | 11 | NA | NA | NA | 1 |  |
| Han et al. | 91 | 0 | 0 | 0 | 0 | 0 | 0 | 0 | 0 | NA | NA | NA | NA |  |
| Kilani et al. | 61 | 4 | NA | 0 | 0 | 0 | 6 | 0 | 5 | NA | 0 | NA | 5 |  |
| Fakiri et al. | 74 | 2 | 6 | 1 | NA | NA | 32 | 22 | 22 | 9 | NA | NA | NA |  |
| Danah et al. | 134 | 11 | 14 | NA | NA | NA | NA | NA | NA | NA | NA | NA | NA |  |
| Mamishi et al. | 24 | NA | NA | 5 | NA | 22 | 4 | 4 | 4 | NA | NA | NA | 21 |  |

CRP, C-reactive protein; LDH, lactate dehydrogenase; AST, aspartate transaminase; ALT, alanine transaminase; CK-MB, creatine kinase MB; ESR, erythrocyte sedimentation rate; NA, not available, not reported.
