# Supplementary material for: Clinical, laboratory, and imaging features of pediatric COVID-19: A systematic review and meta-analysis
Source: Medicine (Baltimore). 2021 Apr 16;100(15):e25230. doi: 10.1097/MD.0000000000025230 (PMC8052054; doi:10.1097/MD.0000000000025230)
Supplement: Supplemental Digital Content [file medi-100-e25230-s010.docx]

**Table S5.** Imaging characteristics, contact history, and severe cases of pediatric COVID-19 patients of the included studies.

| Author | N | Imaging (N) |  |  |  | Contact history | Severe |
| --- | --- | --- | --- | --- | --- | --- | --- |
|  |  | Ground-glass opacity | Bilateral lesions | Unilateral lesion | Normal |  |  |
| Dong et al. | 728 | NA | NA | NA | NA | NA | 21 |
| Wang et al. | 31 | NA | NA | NA | 16 | 22 | 0 |
| Cai et al. | 10 | NA | 0 | 4 | 6 | 8 | NA |
| Chen et al. | 12 | NA | NA | NA | 2 | 12 | 0 |
| Du et al. | 14 | NA | 6 | 5 | NA | NA | 0 |
| Feng et al. | 15 | 7 | NA | NA | 6 | 15 | 0 |
| Bo Li et al. | 22 | 3 | 15 | 5 | 2 | NA | NA |
| Xu et al. | 10 | 5 | NA | NA | NA | 10 | 0 |
| Qiu et al. | 36 | NA | NA | NA | NA | 32 | 0 |
| Tan et al. | 10 | 5 | NA | NA | 5 | 10 | NA |
| Xia et al. | 20 | 12 | 10 | 6 | 4 | NA | NA |
| Zheng et al. | 25 | NA | 11 | 5 | 8 | 21 | 2 |
| Zhu et al. | 10 | NA | 2 | 3 | 5 | 7 | NA |
| Lu et al. | 171 | 56 | NA | NA | 27 | 156 | 3 |
| Li et al. | 5 | 3 | 0 | 3 | 2 | NA | NA |
| Liu et al. | 4 | 1 | 1 | 2 | 1 | 4 | NA |
| Shen et al. | 9 | 2 | 0 | 2 | 7 | 9 | 0 |
| Liang et al. | 9 | NA | NA | NA | 5 | 9 | 0 |
| Xie et al. | 13 | 2 | NA | NA | 9 | 9 | 0 |
| Zhou et al. | 9 | 1 | 4 | 4 | 1 | 9 | NA |
| Han et al. | 7 | NA | NA | NA | 2 | 7 | NA |
| Ma et al. | 50 | 29 | 9 | 16 | 7 | 50 | 2 |
| Korkmaz et al. | 81 | 3 | NA | NA | 24 | 73 | NA |
| Hua et al. | 30 | 7 | 5 | 2 | 23 | 29 | NA |
| Wu et al. | 74 | 9 | 16 | 21 | 37 | 65 | 1 |
| Du et al. | 182 | 51 | 57 | 73 | 52 | 178 | 4 |
| Wu et al. | 148 | 51 | 34 | 51 | NA | NA | NA |
| Parri et al. | 130 | 17 | NA | NA | 15 | 70 | 20 |
| Ma et al. | 216 | NA | 47 | 68 | 101 | 193 | 0 |
| Kilani et al. | 61 | 3 | 1 | 3 | 4 | NA | NA |
| Han et al. | 91 | NA | NA | NA | NA | 87 | 2 |
| Pablo et al. | 91 | 21 | NA | NA | 2 | NA | 9 |
| Afshin et al. | 27 | 8 | 9 | 7 | 9 | NA | NA |
| Fakiri et al. | 74 | NA | NA | NA | NA | 74 | 0 |
| Danah et al. | 134 | NA | NA | NA | NA | 130 | 0 |
| Mamishi et al. | 24 | 7 | NA | NA | 2 | NA | NA |

NA, not available, not reported
